# Multi-level assessment of chronic toxicity of the insect growth regulator lufenuron in the non-target aquatic organism Daphnia magna

**DOI:** 10.1007/s10653-026-03279-5

**Published:** 2026-06-08

**Authors:** Hatice Dane, Özkan Aksakal, Turgay Şişman, Cihan Gür, Ömercan Alat

**Affiliations:** 1 Department of Biology, Faculty of Science, Atatürk University, Erzurum, Turkey; 2 Department of Molecular Biology and Genetics, Faculty of Science, Atatürk University, Erzurum, Turkey; 3 Department of Medical Laboratory Techniques, Vocational School of Health Services, Atatürk University, Erzurum, Turkey; 4 Department of Veterinary Biochemistry, Faculty of Veterinary Medicine, Atatürk University, Erzurum, Turkey

**Keywords:** Lufenuron, *Daphnia magna*, Detoxification, Oxidative stress, Gene expression

## Abstract

Lufenuron, a benzoylurea insect growth regulator, is widely used against various pest species. Despite its broad application, multi-level studies addressing the chronic and sublethal effects of lufenuron on *Daphnia magna* remain limited. In this study, we evaluated the physiological, biochemical, and transcriptional responses of *D. magna* exposed to three lufenuron concentrations (0.01, 0.025, and 0.06 µg/L) over 21 days. The 48-h EC50 was determined as 0.383 µg/L. Chronic exposure was associated with decreased survival, body length, molting frequency, and heart rate. Reproductive performance was also impaired, as shown by a reduced total number of offspring per female and delayed first brood. Biochemical analyses showed increased MDA levels and elevated SOD and CAT activities, along with decreased GST activity. Transcriptional analyses revealed significant induction of Nrf2, upregulation of SOD and CAT, suppression of GST at low concentrations, and significant upregulation of CYP360A8, CYP4, HR96P, and P-gp, while CYP314 expression remained unchanged. In addition, VTG expression increased, whereas CUT and DMRT expression decreased, indicating modulation of genes associated with reproduction and development. These results suggest that chronic lufenuron exposure can adversely affect multiple biological endpoints in *D. magna* and highlight the importance of considering sublethal effects in ecological risk assessment.

**Graphical abstract:**

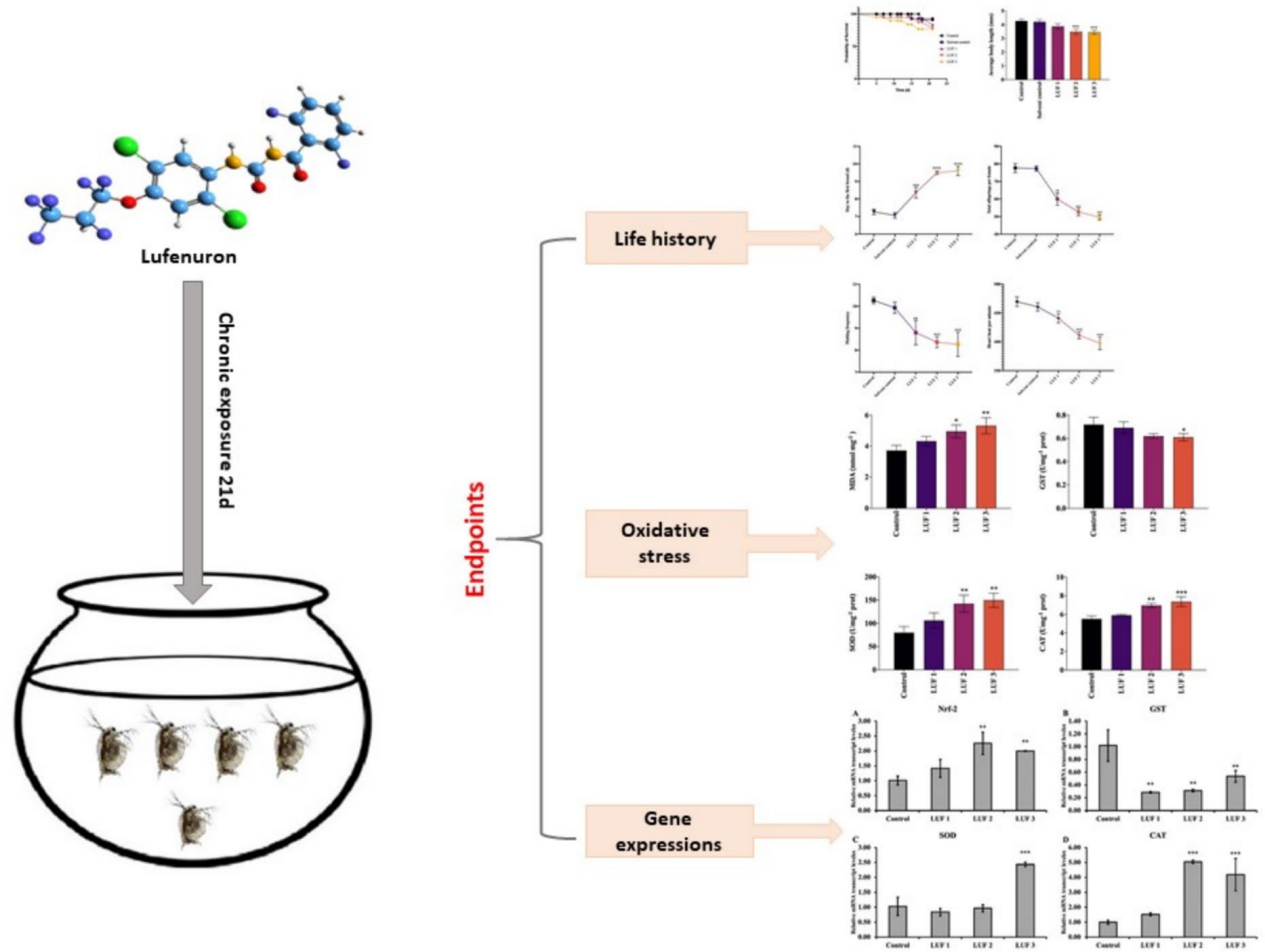

**Supplementary Information:**

The online version contains supplementary material available at 10.1007/s10653-026-03279-5.

## Introduction

Chemical insecticides are extensively used in agricultural, veterinary, and aquaculture practices to control pest and parasite species. However, their continuous application increases the likelihood of environmental contamination, particularly in freshwater ecosystems where residues may enter through agricultural runoff, leaching, effluent discharge, or sediment-associated transport (Brock et al., 2018; Khan et al., 2025; Liang et al., 2024; Saeed et al., 2025). Among modern insecticides, insect growth regulators (IGRs) have received increasing attention because they differ from conventional neurotoxic insecticides by targeting developmental processes such as molting, chitin synthesis, and endocrine-regulated growth. Although this selective mode of action may reduce toxicity to some non-target vertebrates, it can pose a particular risk to aquatic arthropods, whose growth and reproduction depend on successful molting and cuticle formation (Douris et al., 2016; Junquera et al., 2019; Zhang et al., 2022).

Lufenuron is a benzoylurea-type IGR widely used in crop protection, veterinary medicine, and aquaculture. Its primary mode of action is associated with the inhibition of chitin biosynthesis, which can impair exoskeleton formation, disrupt molting, and negatively affect development and reproduction in arthropods (Douris et al., 2016; Ma et al., 2024; Zhang et al., 2022). Recent studies have further shown that lufenuron exposure may alter growth, reproductive performance, oxidative balance, and detoxification-related responses in different organisms, indicating that its biological effects may extend beyond chitin synthesis inhibition alone (Al-Saeed et al., 2023; Ghelichpour et al., 2020; Khan et al., 2025; Saeed et al., 2025). In addition, the low water solubility and high adsorption potential of lufenuron suggest that it may partition into sediments and suspended particles, thereby increasing the likelihood of chronic exposure for aquatic invertebrates living in or near sediment-associated habitats (Brock et al., 2018; EFSA, 2017; FAO, 2008).

Aquatic invertebrates are especially relevant for evaluating the ecological risks of IGRs because many of them possess chitin-containing structures and undergo repeated molting throughout their life cycle. *Daphnia magna* is one of the most widely used freshwater model organisms in ecotoxicology due to its short life cycle, parthenogenetic reproduction, high sensitivity to pollutants, and ecological importance in aquatic food webs (OECD, 2012; Reilly et al., 2023). The OECD 211 reproduction test is specifically designed to assess chronic effects on *D. magna* reproductive output over 21 days, making this species suitable for investigating long-term sublethal toxicity. In addition to apical endpoints such as survival, growth, molting, and reproduction, biochemical and molecular biomarkers can provide mechanistic insight into oxidative stress, detoxification processes, and transcriptional responses associated with contaminant exposure (Barata et al., 2007; Liu et al., 2022; Wang et al., 2016).

Despite the recognized aquatic toxicity of lufenuron, knowledge regarding its chronic and sublethal effects in *D. magna* remains limited. Furthermore, the available literature on lufenuron in aquatic environments remains fragmented, with relatively few studies directly addressing its use and effects in aquatic systems (Burridge & Holmes, 2023). Most existing data are derived either from regulatory toxicity endpoints for *D. magna* or from studies on fish, benthic arthropods, birds, and target pest species, rather than from chronic mechanistic assessments in planktonic microcrustaceans (Brock et al., 2018; FAO, 2008; Ghelichpour et al., 2020; Khan et al., 2025; Ling et al., 2025; Ma et al., 2024; Saeed et al., 2025). Therefore, the mechanisms underlying chronic lufenuron toxicity in *D. magna* remain insufficiently understood. Publicly available regulatory data report high sensitivity of *D. magna* to lufenuron, including acute EC50 values in the low µg/L range and a 21-day NOEC of 0.00010 µg/L; however, these data mainly provide conventional regulatory endpoints and do not explain the underlying physiological, biochemical, or transcriptional responses (FAO, 2008). Studies on other benzoylurea IGRs, such as diflubenzuron, indicate that chitin synthesis inhibitors can affect oxidative stress, chitin-related processes, and reproductive outcomes in *D. magna*, further supporting the need for a focused evaluation of lufenuron in this model organism (Karimova et al., 2025).

Therefore, the present study aimed to evaluate the chronic toxicity of lufenuron in *D. magna* using an integrated approach combining physiological endpoints (survival, reproduction, development), biochemical biomarkers [malondialdehyde (MDA), superoxide dismutase (SOD), catalase (CAT), and glutathione S-transferase (GST)], and transcriptional responses related to detoxification, oxidative stress, reproduction, and development. Based on the chitin synthesis-inhibiting mode of action of lufenuron, we hypothesized that chronic exposure would impair molting, growth, and reproduction in *D. magna*, and that these organism-level effects would be accompanied by oxidative stress and modulation of detoxification-, antioxidant-, and development-related gene expression. By investigating sub-cellular to whole-organismal responses, this study seeks to provide further insight into the multi-level toxicity profile of lufenuron in a non-target freshwater microcrustacean. The findings of this research aim to characterize the multi-level ecotoxicological responses of *D. magna* to chronic exposure, contributing to refined environmental risk assessments and supporting the development of more sustainable and ecologically responsible pest management strategies.

## Materials and methods

### Test organism

The *D. magna* used in the study were obtained from a culture established in our laboratory for > 1.5 years, in accordance with OECD (2012) recommendations. The light/dark, temperature, pH, and dissolved oxygen concentration conditions for the organisms were 16:8 h, 20 ± 2 °C, 7.5 ± 0.5, and 6.0 ± 0.5 mg/L, respectively. A *Chlorella* sp. suspension (2 × 10^5^ cells/mL) was used as food, and *Daphnia* media was renewed twice a week.

### Test chemical

Lufenuron {1-[2,5-Dichloro-4-(1,1,2,3,3,3-hexafluoropropoxy)phenyl]-3-(2,6-difluorobenzoyl)urea} (CAS number: 103055–07-8) as analytical standards was obtained from Sigma-Aldrich (Germany). For the solvent control group, the insecticide was dissolved in acetone (analytical grade). Test solutions were prepared at nominal concentrations and renewed daily throughout both the acute and 21-day chronic exposure periods. Analytical verification of lufenuron concentrations was performed by LC–MS using freshly prepared, 0 h, and 24-h-aged exposure media. Measured concentrations ranged from 87.0% to 95.8% of nominal values and therefore remained within the ± 20% acceptance range recommended by OECD 211 for semi-static exposure systems. Accordingly, nominal concentrations were used for reporting and statistical analysis. Detailed measured concentrations are provided in Table S1.

### Acute toxicity test

The acute toxicity of lufenuron to *D. magna* was determined according to OECD Test Guideline 202. Test concentrations of 0.01, 0.025, 0.06, 0.15, 0.35, 0.90, and 2.25 μg/L were selected based on preliminary experiments and available literature data for lufenuron (Khan et al., 2025). For each concentration, one 250 mL glass beaker containing 200 mL of test solution and 10 neonates younger than 24 h was used in each experimental run. Control and solvent control groups were prepared under the same conditions, with the solvent control containing 0.01% acetone, v/v. Neonates were not fed during the 48-h exposure period. The beaker was defined as the experimental unit, and immobilization percentage was calculated for each beaker based on the number of immobile neonates after 48 h. The acute toxicity assay was conducted in three independent experimental runs using newly prepared test solutions and neonates from different culture cohorts, resulting in three beaker-level replicates per concentration. At the end of 48 h, individuals unable to swim within 15 s after gentle agitation of the beaker were recorded as immobile. The 48-h EC50 value and its 95% confidence limits were calculated using probit analysis in IBM SPSS Statistics version 21.0 (IBM Corp., Armonk, NY, USA). A heterogeneity factor was used in the confidence limit estimation.

### Chronic toxicity test

The chronic toxicity of lufenuron on *D. magna* was conducted in accordance with OECD 211 (OECD, 2012) guidelines, and the concentrations used in the test were selected based on preliminary acute toxicity tests and corresponded to sublethal fractions of the 48 h EC_50_. Concentrations were determined as 0 (control), 0.01 µg/L (LUF 1), 0.025 µg/L (LUF 2), and 0.06 µg/L (LUF 3), respectively. Environmental monitoring and regulatory assessments indicate that lufenuron occurs in surface waters at concentrations ranging from a few nanograms to several tens of nanograms per liter (ng/L). According to evaluations by the U.S. Environmental Protection Agency (EPA) and the European Food Safety Authority (EFSA), predicted environmental concentrations (PECs) in surface waters following agricultural or veterinary use range between 0.001 and 0.06 µg/L (EFSA, 2017; US EPA, 2017). Due to its low water solubility ( ≈ 0.046 mg/L) and high adsorption coefficient (Koc ≈ 4 × 10^4^ mL/g), lufenuron rapidly partitions to sediments and suspended particles rather than remaining in the water column (Brock et al., 2018). Therefore, the exposure concentrations selected in this study (0.01–0.06 µg/L) are within the range of predicted environmental exposure scenarios and can be considered relevant for assessing chronic sublethal effects (FAO, 2008; Government of Canada, 2023; Rafaela Leão Soares et al., 2016). For the chronic toxicity experiment, female neonates (< 24 h old) were individually exposed to lufenuron in 50 mL glass beakers containing 20 mL of M4 medium with the corresponding test solution. Each beaker contained one neonate and was considered one biological replicate for life-history and physiological endpoints. For life-history endpoints, 10 independent beakers were prepared per treatment in each experimental run. The chronic exposure experiment was repeated five times independently using newly prepared test solutions and different neonate cohorts. Additional parallel beakers were established under the same exposure conditions to obtain sufficient biological material for downstream biochemical and gene expression analyses. The biological replicate structure for pooled biochemical and gene expression analyses is described separately below. Test solutions were renewed daily throughout the 21-day exposure period to maintain stable exposure conditions, considering the low water solubility and high adsorption potential of lufenuron. Animals were fed daily with a *Chlorella* sp. suspension at a concentration of 2 × 10^5^ cells/mL during the chronic exposure period. At the end of the 21-day exposure, physiological parameters, biochemical assays, and gene transcription analyses were performed.

### Physiological parameters

Physiological measurements were performed on individuals that survived the exposure. The number of days until the first brood, the total number of offspring per female, and the molting frequency were recorded through daily observations.

Survival was recorded daily throughout the 21-day exposure period. Because each beaker contained one individual *D. magna*, survival status was recorded for each replicate beaker as alive or dead at each observation time. Survival probability for each treatment group was calculated as the number of surviving individuals at a given observation time divided by the initial number of individuals in that treatment group across replicate beakers. Survival values were expressed as percentages and plotted over time using GraphPad Prism to generate survival curves for each treatment group. Molting frequency was defined as the average number of molts per individual during the 21-day exposure period. For body length and heart rate measurements, 10 surviving females per treatment were randomly selected from the life-history beakers at the end of exposure. Body length was determined by measuring the distance from the top of the head to the base of the tail spine under a LEICA DM750 microscope using LEICA LAS EZ image processing software. The microscope imaging system was calibrated with a stage micrometer before measurements. For each individual, body length was measured twice by the same trained observer, and the mean value was used for statistical analysis. For heart rate measurement, individual daphnids were transferred onto a microscope slide with 50 μL of exposure medium and gently immobilized using cotton fibers to restrict excessive movement. Heart rate was recorded for 1 min under the LEICA DM750 microscope and expressed as beats per minute (bpm). Measurements were performed by the same observer under identical magnification and temperature conditions to minimize observer-related variability.

### Biochemical analysis

Following the 21-day exposure period, surviving adult *D. magna* individuals from the same treatment group were pooled to obtain sufficient biomass for biochemical analyses. Each biological replicate consisted of a pool of 30 individuals collected from independent individual beakers exposed under the same treatment condition. Five independent pooled biological replicates were prepared per treatment. Thus, the pooled sample, not the individual organisms within the pool, was treated as the unit of statistical analysis. Samples were briefly rinsed with ice-cold PBS and homogenized in 0.1 M ice-cold PBS, pH 7.4, using a tissue homogenizer under cold conditions. The homogenates were centrifuged at 9000 × g for 20 min at 4 ℃, and the resulting supernatants were used for biochemical analyses.

Malondialdehyde (MDA) levels, glutathione S-transferase (GST), superoxide dismutase (SOD), and catalase (CAT) activities were determined using commercial assay kits (Sigma-Aldrich MAK 568 MDA Assay Kit, Sigma-Aldrich MAK 453 GST Assay Kit, Sigma-Aldrich 19,160 SOD Assay Kit, and Sigma-Aldrich CAT 100 Assay Kit) according to the manufacturers’ instructions. Standard calibration curves were generated where applicable, samples were analyzed in technical duplicates, and assay sensitivity, detection range, and intra-assay consistency were checked according to the manufacturer’s specifications.

### Gene expression analysis and quantitative real-time PCR assay

Detoxification-related gene expression analyses included CYP360A8, CYP4, CYP314, HR96P, and P-gp; oxidative stress response included Nrf2, GST, SOD, and CAT; reproduction- and development-associated genes included VTG, CUT, and DMRT. For gene expression analysis, surviving adult *D. magna* individuals from the same treatment group were pooled to obtain sufficient RNA. Each biological replicate consisted of a pool of 10 individuals collected from independent individual beakers exposed under the same treatment condition. Three independent pooled biological replicates were prepared per treatment. For each biological replicate, qRT-PCR reactions were performed in triplicate from the same cDNA sample; these triplicate reactions were considered technical replicates. The mean Ct value of the technical triplicates was used for relative gene expression analysis. Statistical analyses were performed using biological replicate pools, not individual organisms within the pools or technical qPCR replicates. Total RNA was extracted from *D. magna* using QIAzol Lysis Reagent (79.306; Qiagen) according to the manufacturer’s protocols. The quality and quantity of the extracted RNAs were measured using a Nanodrop ND spectrophotometer. cDNA from RNA was obtained using the iScript cDNA Synthesis Kit (BIO-RAD, USA). 1 μL of cDNA, 12.5 μL of QuantiTect SYBR Green PCR Master Mix (204,143; Qiagen), 10.5 μL of RNase-free water, and 1 μL of gene-specific primers (Table S2) were added to a reaction tube. The expression of target genes was measured using the Rotor-gene Q Real-Time PCR Detection System (Qiagen, Basel, Switzerland). For the quantitative RT-PCR amplification procedure, pre-denaturation was performed at 95 ℃ for 3 min, followed by 40 cycles of denaturation at 95 ℃ for 10 s and annealing and extension at 60 ℃ for 1 min. β-actin was used as the reference gene for normalization. To assess its suitability under the present experimental conditions, β-actin Ct values were compared across all control and lufenuron-exposed groups using one-way ANOVA. No significant differences were detected among groups, p > 0.05. In addition, melting curve analysis was performed after each qRT-PCR run to verify amplification specificity. A single sharp melting peak with a consistent melting temperature was observed across samples, indicating specific amplification without detectable primer-dimer formation. The β-actin Ct stability data and representative melting curve are provided in Supplementary Table S3 and Fig. S1. Relative gene expression results were analyzed using the 2^−△△CT^ method.

### Statistical analysis

Data were analyzed using one-way analysis of variance (ANOVA) in GraphPad Prism and IBM SPSS Statistics. Prior to analysis, the assumptions of normality and homogeneity of variance were evaluated. When significant differences were detected, Tukey’s HSD post-hoc test was applied to identify pairwise differences among groups and to control Type I error inflation due to multiple pairwise comparisons within each endpoint. Data are presented as mean ± SD. Statistical significance was reported at p < 0.05, p < 0.01, p < 0.001, and p < 0.0001. Acute immobilization data were analyzed separately using probit analysis in IBM SPSS Statistics version 21.0 to estimate the 48-h EC50 value and its 95% confidence limits. Because no global multiple-testing correction was applied across all measured endpoints, statistically significant findings were interpreted together with their biological consistency across related physiological, biochemical, and transcriptional responses.

## Results

Acute exposure to lufenuron for 48 h caused changes in swimming behavior in *D. magna*. At the highest concentration tested, immobility was observed in all individuals. The 48-h EC_50_ of lufenuron was calculated as 0.383 μg/L (95% CL: 0.262—0.587).

**Fig. 1:**
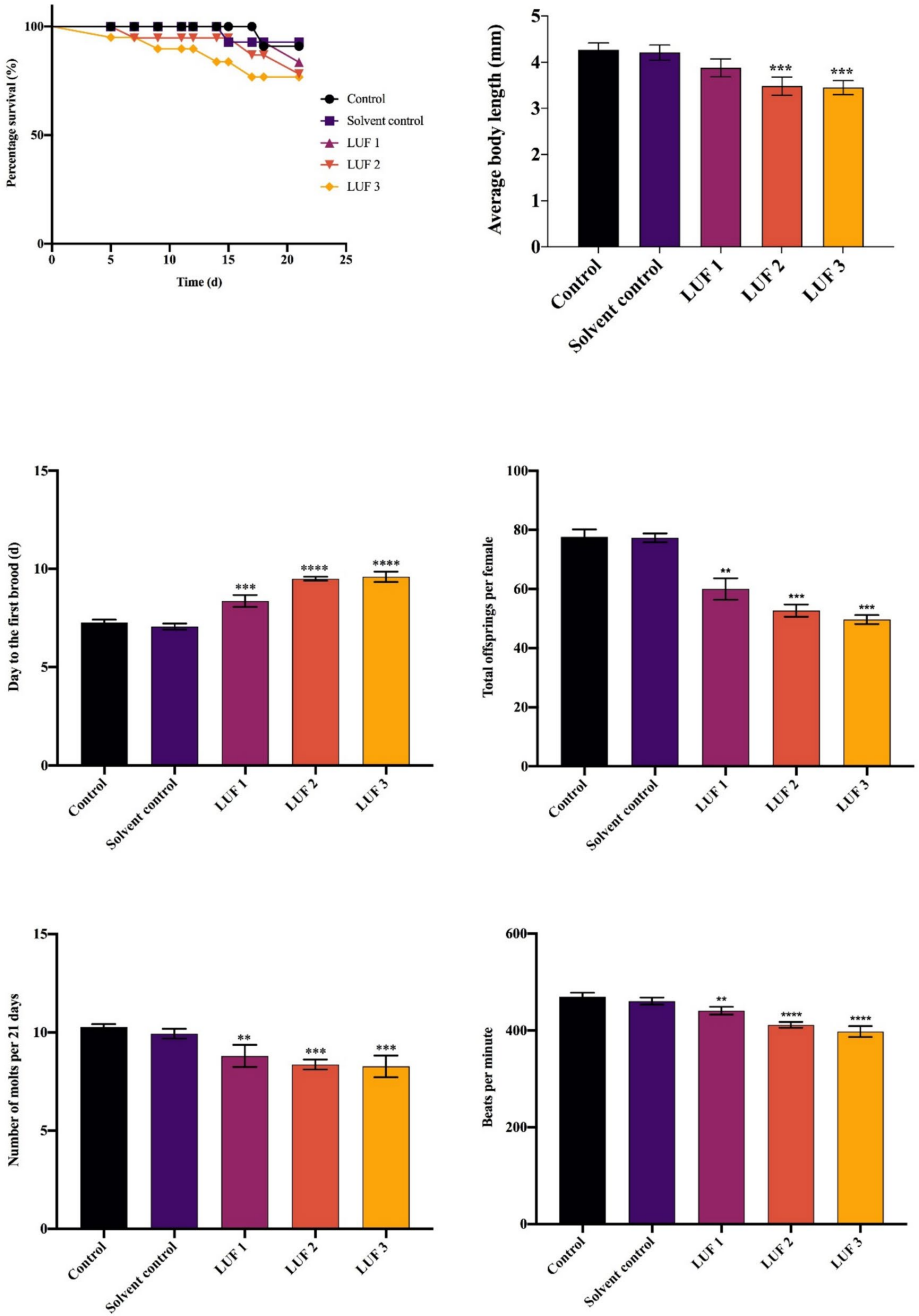
Effects of chronic lufenuron exposure on physiological parameters of *D. magna*, including survival (%), body length (mm), heart rate (beats per minute, bpm), molting frequency (number of molts per 21 days), time to first brood (days), and total number of offspring per female. Data are presented as mean ± SD. LUF 1: 0.01 μg/L, LUF 2: 0.025 μg/L, LUF 3: 0.06 μg/L. Asterisks indicate statistically significant differences compared to the control group (*p < 0.05, **p < 0.01, ***p < 0.001, ****p < 0.0001)

Chronic exposure to lufenuron significantly affected physiological responses in *D. magna* (Fig. 1). Survival decreased with increasing lufenuron exposure, with significant reductions observed at 0.025 and 0.06 µg/L. While the control group exhibited high survival throughout the test period, significant decreases were observed at 0.025 and 0.06 µg/L, with the most pronounced reduction at 0.06 µg/L, indicating reduced survival under chronic exposure conditions.

Body length was significantly reduced in lufenuron-exposed groups, particularly at 0.025 and 0.06 µg/L. Control animals reached an average length of approximately 4.3 mm. In contrast, individuals exposed to 0.025 and 0.06 µg/L were significantly smaller, with mean lengths around 3.5 mm, corresponding to an approximately 19% reduction relative to the control (p < 0.001), indicating growth inhibition.

Reproductive output was also negatively affected. The time to first brood was significantly delayed at all exposure concentrations, extending from approximately day 7 in the control to day 9 at 0.01 µg/L and day 10 at 0.025 and 0.06 µg/L. Relative to the control, these changes corresponded to an approximately 29% delay at 0.01 µg/L and a 43% delay at both 0.025 and 0.06 µg/L. In parallel, the total number of offspring per female decreased significantly from an average of 78 in the control to 60, 50, and 48 at 0.01, 0.025, and 0.06 µg/L, respectively. These values corresponded to approximately 23%, 36%, and 39% reductions relative to the control, respectively (p < 0.01–0.001).

Molting frequency was significantly reduced following exposure to higher concentrations of lufenuron. Over the 21-day exposure, control animals exhibited 10.1 ± 0.3 molts, whereas significant reductions were observed at 0.025 µg/L (8.2 ± 0.4, p < 0.01) and 0.06 µg/L (8.1 ± 0.3, p < 0.001), indicating a slower molting cycle under lufenuron exposure. These reductions corresponded to approximately 19% and 20% decreases relative to the control.

Heart rate also declined significantly with increasing concentrations. Control daphnids had a mean heart rate of approximately 440 beats per minute, which was significantly reduced to 395 bpm at 0.025 μg/L and 375 bpm at 0.06 μg/L. These values corresponded to approximately 10% and 15% decreases relative to the control, respectively (p < 0.001), indicating altered cardiac physiological performance under chronic exposure.

**Fig. 2:**
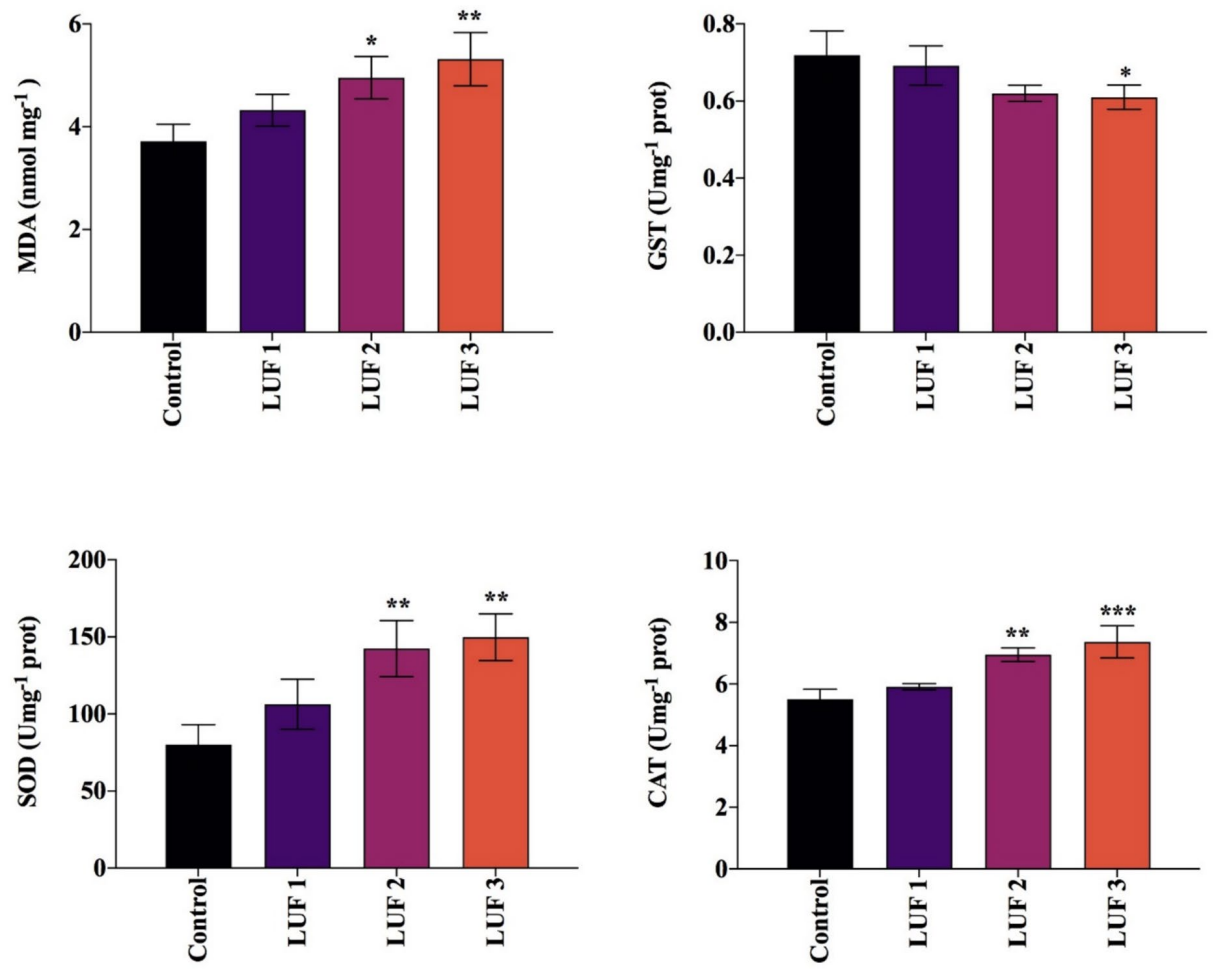
Effects of chronic lufenuron exposure on oxidative stress biomarkers in *D. magna*, including MDA levels (nmol/mg protein), SOD, CAT, and GST activities (U/mg protein). Data are presented as mean ± SD. LUF 1: 0.01 μg/L, LUF 2: 0.025 μg/L, LUF 3: 0.06 μg/L. Asterisks indicate statistically significant differences compared to the control group (*p < 0.05, **p < 0.01, ***p < 0.001)

Lufenuron exposure induced oxidative stress in *D. magna*, as evidenced by elevated malondialdehyde (MDA) levels and altered antioxidant enzyme activities (Fig. 2). MDA content increased significantly at 0.025 and 0.06 µg/L compared with the control (p < 0.05 and p < 0.01, respectively), corresponding to approximately 30–45% increases and indicating enhanced lipid peroxidation. Glutathione S-transferase (GST) activity showed a decreasing trend with increasing lufenuron concentrations, with an approximately 15% reduction observed at 0.06 µg/L (p < 0.05). In contrast, superoxide dismutase (SOD) activity increased by approximately 75–85% at 0.025 and 0.06 µg/L, while catalase (CAT) activity increased by approximately 25–35%, consistent with activation of antioxidant-related responses.

**Fig. 3:**
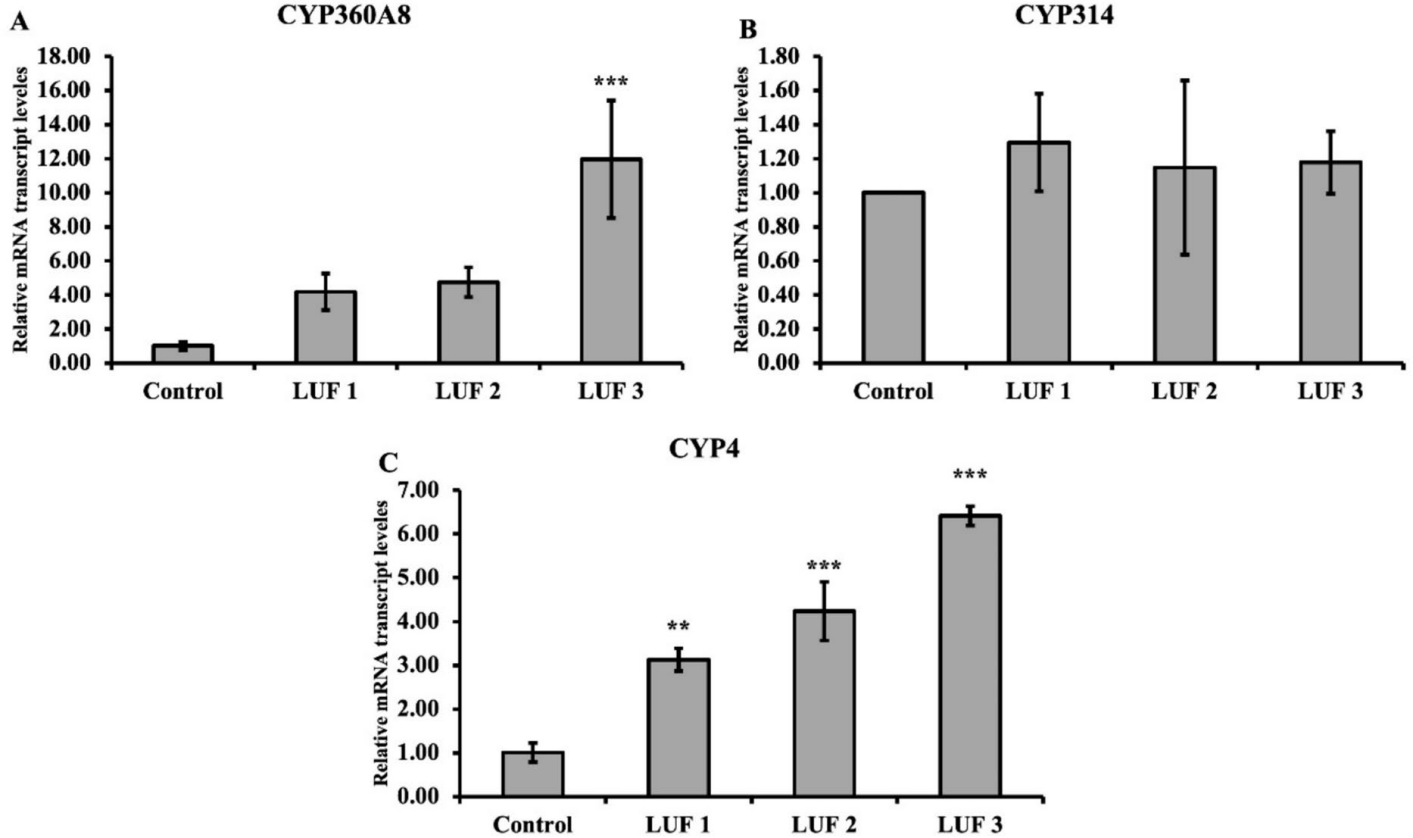
Relative expression levels of cytochrome P450-related genes (CYP360A8, CYP4, CYP314) in *D. magna* following chronic lufenuron exposure. Gene expression was quantified using the 2^−ΔΔCt^ method and normalized to β-actin. Data are presented as mean ± SD. LUF 1: 0.01 μg/L, LUF 2: 0.025 μg/L, LUF 3: 0.06 μg/L. Asterisks indicate statistically significant differences compared to the control group (*p < 0.05, **p < 0.01, ***p < 0.001)

**Fig. 4:**
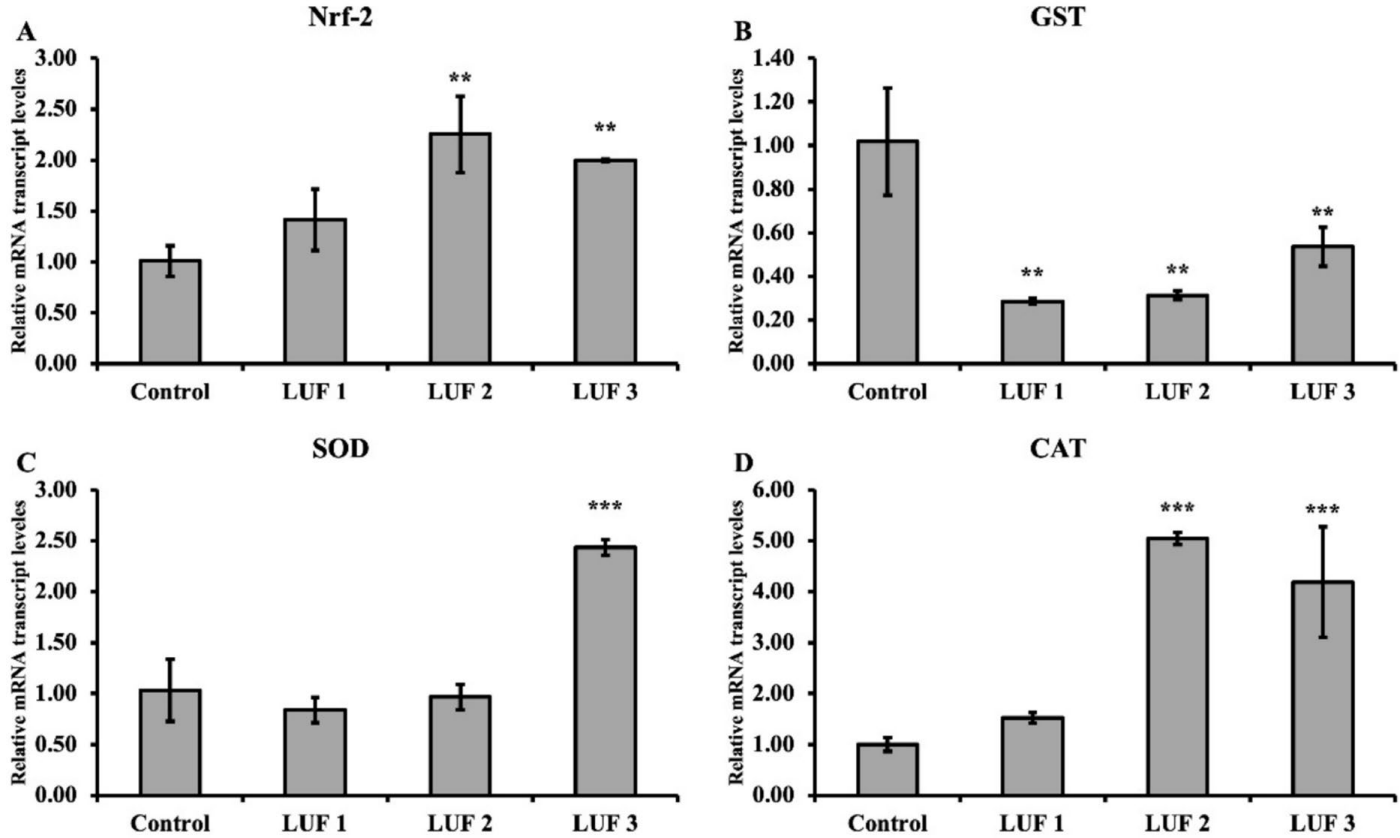
Relative expression levels of oxidative stress-related genes (Nrf2, SOD, CAT, GST) in *D. magna* following chronic lufenuron exposure. Gene expression was quantified using the 2^−ΔΔCt^ method and normalized to β-actin. Data are presented as mean ± SD. LUF 1: 0.01 μg/L, LUF 2: 0.025 μg/L, LUF 3: 0.06 μg/L. Asterisks indicate statistically significant differences compared to the control group (*p < 0.05, **p < 0.01, ***p < 0.001)

**Fig. 5:**
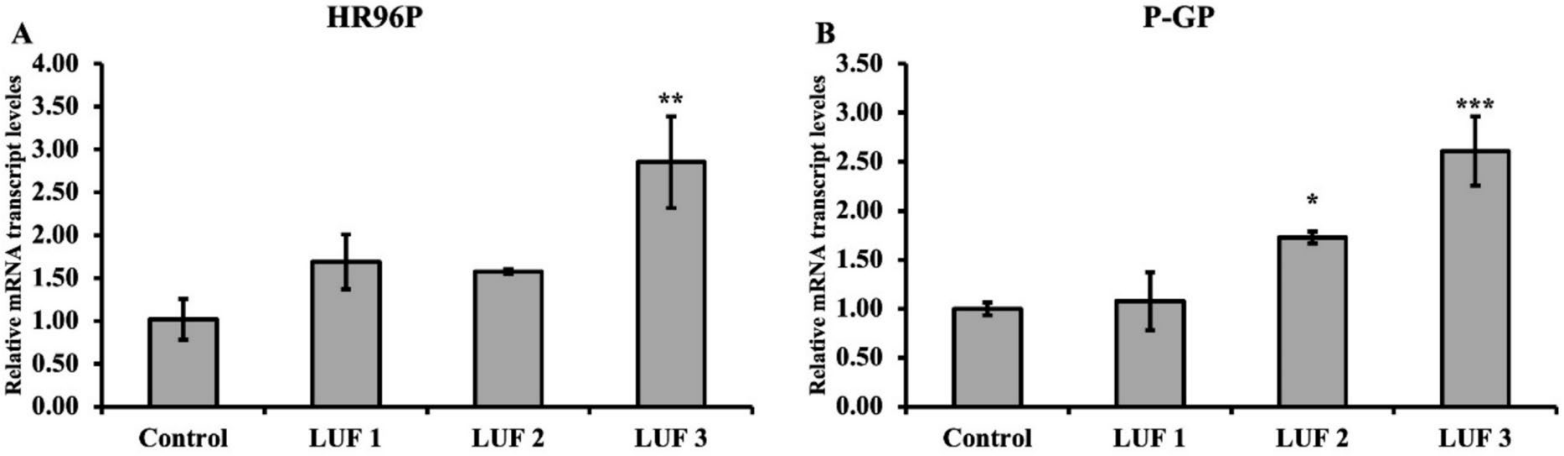
Relative expression levels of detoxification-related genes (HR96P, P-gp) in *D. magna* following chronic lufenuron exposure. Gene expression was quantified using the 2^−ΔΔCt^ method and normalized to β-actin. Data are presented as mean ± SD. LUF 1: 0.01 μg/L, LUF 2: 0.025 μg/L, LUF 3: 0.06 μg/L. Asterisks indicate statistically significant differences compared to the control group (*p < 0.05, **p < 0.01, ***p < 0.001)

**Fig. 6:**
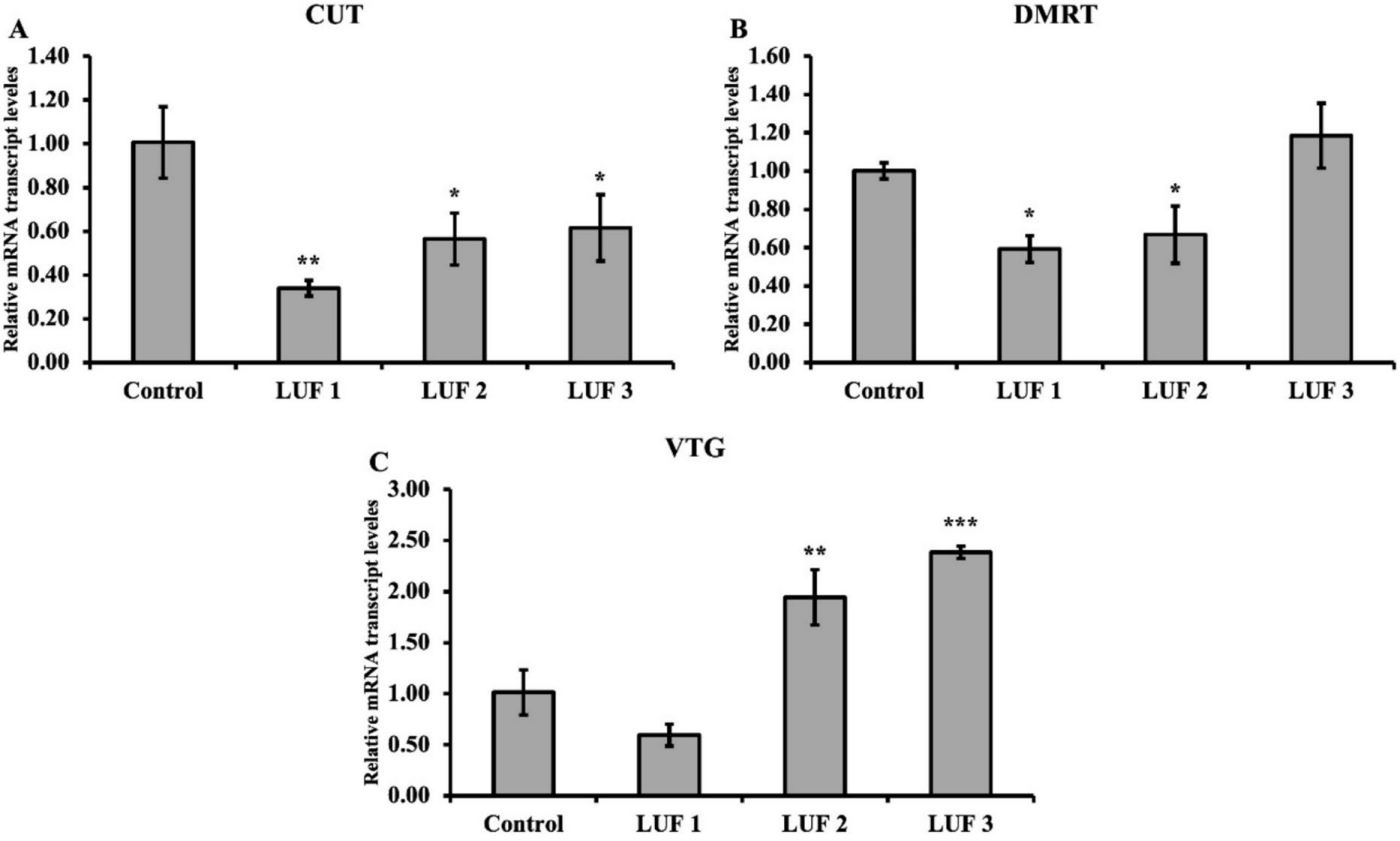
Relative expression levels of genes associated with reproduction and development (VTG, CUT, DMRT) in *D. magna* following chronic lufenuron exposure. Gene expression was quantified using the 2^−ΔΔCt^ method and normalized to β-actin. Data are presented as mean ± SD. LUF 1: 0.01 μg/L, LUF 2: 0.025 μg/L, LUF 3: 0.06 μg/L. Asterisks indicate statistically significant differences compared to the control group (*p < 0.05, **p < 0.01, ***p < 0.001)

Exposure to lufenuron significantly altered the expression of multiple genes associated with detoxification, oxidative stress response, endocrine signaling, and development in *D. magna* (Fig. 3, 4, 5 and 6). Cytochrome P450-related genes responded differently to lufenuron. CYP360A8 expression was markedly upregulated at 0.06 µg/L, reaching approximately 12-fold of the control level (p < 0.001). CYP4 expression increased across lufenuron-exposed groups, with approximately 3-, 4-, and sixfold induction at 0.01 µg/L, 0.025 μg/L, and 0.06 μg/L, respectively. In contrast, CYP314 transcript levels remained close to control levels across all exposure groups.

Regarding oxidative stress-related pathways, Nrf2 expression increased by approximately twofold at 0.025 and 0.06 µg/L (p < 0.01). GST gene expression was suppressed, particularly at 0.01 and 0.025 µg/L, with approximately 70% reductions. SOD expression increased approximately 2.4-fold at 0.06 μg/L, while CAT expression showed a stronger induction, reaching approximately 4–fivefold higher levels at 0.025 and 0.06 µg/L.

HR96P and P-gp were also induced, with the strongest responses at 0.06 μg/L, reaching approximately 2.8-fold and 2.6-fold of the control levels, respectively.

Expression of genes associated with reproduction and development also showed marked changes. CUT expression decreased across all treatments, with reductions of approximately 40–65% relative to the control (p < 0.01). DMRT expression was reduced by approximately 35–40% at 0.01 and 0.025 µg/L but returned to near-control levels at 0.06 µg/L. VTG expression increased by approximately twofold at 0.025 μg/L and 2.4-fold at 0.06 μg/L, suggesting altered transcriptional regulation of reproduction-associated pathways.

## Discussion

The present study shows that chronic lufenuron exposure was associated with changes in physiological, biochemical, and transcriptional responses in the non-target organism *D. magna*. The results indicate a multi-level response profile involving altered oxidative stress biomarkers, detoxification-related transcriptional responses, and adverse changes in survival, growth, molting, and reproduction. To support the environmental relevance of the selected exposure concentrations, the nominal concentrations used in the present study, 0.01–0.06 µg/L, corresponding to 10–60 ng/L, were directly compared with available environmental monitoring data. Recent monitoring across multiple environmental matrices in agricultural systems reported lufenuron concentrations ranging from 0.161 to 441 ng/g in soils, up to 1920 ng/g in ditch sediments, and up to 52.5 ng/L in ditch water (Qiu et al., 2024). Lufenuron has also been detected in sediments near Canadian Atlantic salmon aquaculture sites, with reported sediment concentrations up to 92.3 ng/g (Kingsbury et al., 2023). Therefore, the exposure range used in the present study overlaps with or is close to upper-range concentrations reported in runoff-impacted aquatic systems, particularly for the highest concentration, 0.06 µg/L. Moreover, because lufenuron has low water solubility and a high potential for particle-bound transport, sediment and suspended particles may represent important exposure compartments in aquatic environments (PPDB, 2026). Thus, water-column concentrations alone may underestimate the ecological exposure of aquatic invertebrates. These comparisons suggest that the selected concentrations fall within a range that may be environmentally relevant, particularly under agricultural runoff, sediment-associated contamination, or worst-case exposure scenarios.

### Physiological effects

High concentrations of lufenuron significantly reduced the survival of *D. magna*. Regulatory datasets indicate that *D. magna* is highly sensitive to lufenuron. The 48-h EC50 values of lufenuron ranged from 1.1 to 1.3 µg/L for *D. magna* in regulatory datasets (FAO, 2008; PPDB, 2026). In an acute test conducted in the presence of sediment, a higher EC50 value of 4.0 µg/L was reported. In addition, a 21-day reproduction NOEC of 0.10 µg/L has been reported, confirming the high chronic sensitivity of *D. magna* to lufenuron (FAO, 2008; PPDB, 2026). (Manrique Guillén et al., 2018) also identified *D. magna* as the most sensitive species and reported a 48-h LC50 value of 0.05 µg/L and 21-day reproduction NOEC/LOEC values of 0.05/0.1 ng/L, respectively. Nevertheless, the available data collectively indicate that *D. magna* responds to lufenuron in the sub-µg/L to low-µg/L range. The 48-h EC50 value obtained in the present study, 0.383 µg/L, is consistent with the high sensitivity reported in the literature. This value is lower than the 1.1–1.3 µg/L range reported for technical lufenuron, but higher than the 0.05 µg/L value reported by (Manrique Guillén et al., 2018). Such differences may reflect methodological variation among studies, including test material, purity, solvent use, neonate age, water chemistry, and whether nominal or analytically verified concentrations were used. Overall, the agreement in order of magnitude supports the reliability of the acute toxicity estimate obtained in the present study.

Evidence from other microcrustacean and zooplankton communities also confirms the high sensitivity of aquatic arthropods to lufenuron. For example, in outdoor ditch systems treated with lufenuron at a nominal concentration of 3 µg/L, Cyclopoida was reported as the most affected group, followed by *Daphnia gr. galeata*, while indirect effects were observed in *Ceriodaphnia* and rotifers (López-Mancisidor et al., 2008). Sediment-spiked microcosm and laboratory studies have also confirmed the sensitivity of benthic arthropods, such as *Hyalella azteca* and *Chironomus riparius*, to benzoylurea insect growth regulators (Brock et al., 2016, 2018). Moreover, the combined effects of lufenuron and increased temperature have been shown to cause synergistic adverse effects on *Daphnia* sp., Cyclopoida, and Copepoda nauplii (Arenas-Sánchez et al., 2019). A similar sensitivity of cladocerans to benzoylurea-type chitin synthesis inhibitors has also been reported for diflubenzuron; exposure to 0.2–0.8 µg/L diflubenzuron adversely affected survival, growth, reproduction, and chitobiase activity in *D. magna* and *D. pulex* (Duchet et al., 2011). These findings are consistent with the specific mode of action of lufenuron as a chitin synthesis inhibitor that can impair molting and development in arthropods. Overall, the present results and previous literature indicate that lufenuron may pose a risk to non-target freshwater microcrustaceans and other aquatic arthropods even at very low concentrations.

The physiological, biochemical, and transcriptional alterations observed in this study may be associated with the decreased survival recorded at 0.025 and 0.06 µg/L. This finding is consistent with studies suggesting that lufenuron suppresses arthropod populations by disrupting molting and early developmental processes (Douris et al., 2016; Ling et al., 2025). A significant decrease in mean body length indicated growth impairment in *D. magna* following chronic lufenuron exposure. This finding is consistent with studies reporting that lufenuron causes this inhibition by interfering with the synthesis or accumulation of chitin, especially in chitinized structures (Lv et al., 2023, 2022). The delay in first brood and the decrease in total offspring per female indicate reduced reproductive performance under lufenuron exposure. These findings are consistent with studies showing that lufenuron limits reproduction in other organisms (Basal et al., 2020; Lv et al., 2023). Overall, the decreases in survival, growth, and reproduction, critical parameters for population dynamics, indicate that chronic lufenuron exposure may pose a risk to *D. magna* under environmentally relevant exposure scenarios.

Reduced molting frequency and heart rate at 0.025 and 0.06 µg/L indicate impaired molting performance and altered physiological activity. This finding is consistent with a physiological stress response. Because lufenuron acts as a chitin synthesis inhibitor, the reduction in molting frequency may be linked to disruption of chitin synthesis and degradation processes. Lufenuron, as a chitin synthesis inhibitor, has been reported to interfere with the formation and metabolism of epidermal chitin, preventing proper molting (Lv et al., 2022; Ma et al., 2024). Similarly, (Liu et al., 2023) reported that high-dose insecticide exposure decreased heart rate and energy metabolism in *D. magna*.

### Biochemical markers

Biochemical analyses indicated that lufenuron exposure was associated with oxidative stress-related responses in *D. magna*, as evidenced by elevated MDA levels and altered antioxidant enzyme activities. Increased MDA levels are indicative of lipid peroxidation and indicate impaired cellular membrane integrity (Basal et al., 2020). Increased SOD and CAT activities indicate the activation of defense mechanisms for detoxification of increased reactive oxygen species (ROS) (Ghelichpour et al., 2020; Karimova et al., 2025; Zhang et al., 2023). Conversely, decreased GST activity suggests impairment or alteration of GST-mediated detoxification capacity. These findings were also obtained in Japanese quail exposed to lufenuron (Saeed et al., 2025) and in studies addressing high-dose insecticide exposure in *D. magna* (Karimova et al., 2025; Zhang et al., 2023). Moreover, antioxidant-related gene expression profiles, particularly Nrf2, SOD, and CAT, showed changes that were broadly consistent with the biochemical antioxidant responses. However, because the relationships among transcriptional, enzymatic, and physiological endpoints were not quantitatively modeled, these associations should be interpreted cautiously. These results suggest that chronic lufenuron exposure affects multiple response pathways, although direct pathway-level validation was not performed.

### Gene expression patterns

Molecular responses suggest transcriptional modulation of antioxidant-related genes, with increased Nrf2 expression at 0.025 and 0.06 µg/L. Significant suppression of GST gene expression at 0.01 and 0.025 µg/L and partial recovery at 0.06 µg/L suggest a non-monotonic transcriptional response in GST-associated detoxification. Significant upregulation of SOD and CAT genes only at 0.06 µg/L is consistent with the biochemically detected elevated enzyme activities. These findings are consistent with previous studies suggesting that alterations in detoxification and oxidative stress responses are associated with adverse outcomes in *D. magna* (Liu et al., 2022; Wang et al., 2016)*.*

Although a formal correlation analysis between gene expression levels and corresponding enzyme activities was not performed, some concordance was observed between selected transcriptional and biochemical responses. In particular, the upregulation of antioxidant-related genes such as SOD and CAT at higher lufenuron concentrations was generally accompanied by increased activities of the corresponding enzymes, suggesting activation of antioxidant defense responses at both transcriptional and biochemical levels. In contrast, GST showed a more complex pattern, with reduced enzymatic activity and variable transcriptional regulation, indicating that detoxification-related responses may not be directly inferred from mRNA levels alone. Therefore, these observations should be interpreted as descriptive and supportive rather than as evidence of a direct quantitative relationship between gene expression and enzyme activity. It should also be noted that gene expression and enzyme activity measurements were conducted on pooled samples derived from different sets of individuals, which limits the ability to establish direct relationships between these endpoints. Moreover, discrepancies between mRNA expression and enzyme activity may arise due to post-transcriptional and post-translational regulatory processes. Future studies integrating molecular and biochemical measurements from the same biological samples, together with correlation or multivariate analyses, are needed to clarify these relationships more accurately.

The induction of cytochrome P450-related genes, particularly CYP360A8 and CYP4, suggests transcriptional activation of xenobiotic metabolism-associated responses (David et al., 2013). In the study, expression of CYP360A8 and CYP4 was significantly increased, particularly at 0.06 µg/L, while CYP314 transcript levels remained unchanged in all exposure groups. This finding suggests that cytochrome P450-related genes respond differently to lufenuron. This has also been reported in studies demonstrating that adaptive responses to lufenuron do not always involve the entire P450 gene family (Douris et al., 2016; Nascimento et al., 2015). In this study, increases in HR96P and P-gp at 0.025 and 0.06 µg/L suggest transcriptional modulation of genes associated with detoxification regulation and xenobiotic efflux. HR96, found in invertebrates, plays a role in controlling the expression of toxic stress-responsive genes such as GST and P-gp, while P-gp is involved in the energy-dependent efflux of various endogenous compounds and xenobiotics. Significant induction of these genes may represent a compensatory transcriptional response to xenobiotic stress, as previously reported in water fleas exposed to different types of insecticides (Liu et al., 2023).

The suppression of CUT and DMRT genes, together with increased VTG expression, indicates altered expression of genes associated with development and reproduction. CUT is related to cuticle-associated developmental processes, whereas DMRT is involved in sex differentiation and reproductive regulation. Therefore, the downregulation of these genes may be associated with the observed impairment of growth, molting, and reproductive performance. VTG, which encodes vitellogenin, a precursor of yolk proteins in oviparous organisms, was significantly upregulated in the present study. This suggests that lufenuron exposure may affect reproduction-related transcriptional pathways in *D. magna*. However, because hormone levels, receptor activity, and vitellogenin protein levels were not measured, this response should not be interpreted as definitive evidence of endocrine disruption. Previous studies have shown that vitellogenin expression in *D. magna* can be modulated by chemicals affecting ecdysteroid-related pathways. (Hannas et al., 2011) reported that vitellogenin mRNA accumulation may be suppressed by ecdysteroid agonists and induced by anti-ecdysteroid compounds, while (Salesa et al., 2024) reported altered vitellogenin expression following exposure to the insect growth regulator pyriproxyfen. Thus, the VTG response observed here may indicate reproduction-related transcriptional modulation, but further hormonal, receptor-level, or protein-level validation is required to confirm endocrine-specific effects.

### Limitations and uncertainty

Several limitations and sources of uncertainty should be considered when interpreting the present findings. First, although β-actin stability was internally assessed using Ct value comparison and melting curve analysis, the use of a single reference gene remains a limitation compared with validation using multiple reference genes. Second, transcriptional changes were not confirmed at the protein, receptor, or hormonal level; therefore, gene expression responses should not be interpreted as direct functional or endocrine-specific evidence. Third, the relationships among physiological, biochemical, and transcriptional endpoints were interpreted mainly in an associative manner, since quantitative integration methods such as correlation analysis, multivariate modeling, or causal pathway analysis were not performed. In addition, the use of pooled samples for biochemical and qRT-PCR analyses may have reduced individual-level variability, while physiological measurements performed only on surviving individuals may have introduced survivor bias. Finally, although exposure concentrations were analytically verified and compared with available environmental monitoring data, some uncertainty may remain due to the low water solubility and high sorption potential of lufenuron. Therefore, the proposed links among oxidative stress, detoxification responses, reproductive/developmental gene expression, and physiological impairment should be regarded as hypothesis-generating and should be validated in future studies.

## Conclusion

This study evaluated the chronic effects of lufenuron on the non-target freshwater crustacean *D. magna* using physiological, biochemical, and transcriptional endpoints. Chronic exposure to selected sublethal concentrations of lufenuron was associated with reductions in survival, body length, molting frequency, heart rate, and reproductive output, indicating alterations in key life-history traits. In parallel, increased MDA levels, changes in antioxidant enzyme activities, and transcriptional modulation of genes related to oxidative stress, detoxification, reproduction, and development were observed.

Overall, these findings suggest that chronic lufenuron exposure can affect multiple biological responses in *D. magna*, with potential implications for freshwater microcrustaceans under long-term exposure scenarios. The combined physiological and biochemical responses indicate that oxidative stress and altered detoxification capacity may contribute to the observed adverse outcomes. However, transcriptional changes should be interpreted as supportive molecular responses rather than direct evidence of protein-level, hormonal, or functional alterations.

The present study provides a multi-level assessment of lufenuron toxicity in *D. magna* and contributes to the understanding of its potential ecological risk in freshwater environments. Future studies incorporating multiple validated reference genes, protein-level and hormonal measurements, receptor-level analyses, quantitative integration among endpoints, and multigenerational or sediment-associated exposure designs are needed to further clarify the mechanisms and ecological consequences of lufenuron exposure.

## Supplementary Information

Below is the link to the electronic supplementary material.

Supplementary file1 (DOCX 128 KB)

## Data Availability

All data generated or analysed during this study are included in this published article and its supplementary information files.
