# Supplementary material for: Multi-level assessment of chronic toxicity of the insect growth regulator lufenuron in the non-target aquatic organism Daphnia magna
Source: Environ Geochem Health. 2026 Jun 8;48(9):394. doi: 10.1007/s10653-026-03279-5 (PMC13246863; doi:10.1007/s10653-026-03279-5)
Supplement: Supplementary file 1 — Supplementary file1 (DOCX 128 KB) [file 10653_2026_3279_MOESM1_ESM.docx]

**Table S1** Measured concentrations of lufenuron in exposure media

| Nominal concentration (µg/L) | Measured concentration at 0 h (µg/L) | % of nominal at 0 h | Measured concentration at 24 h (µg/L) | % of nominal at 24 h |
| --- | --- | --- | --- | --- |
| 0.01 | 0.0091 | 91.0 | 0.0087 | 87.0 |
| 0.025 | 0.0238 | 95.2 | 0.0229 | 91.6 |
| 0.06 | 0.0575 | 95.8 | 0.0558 | 93.0 |

Analytical verification of lufenuron concentrations was performed using liquid chromatography–mass spectrometry, LC–MS. Samples were collected from freshly prepared exposure media, 0 h, and from 24-h-aged exposure media immediately before renewal. Chromatographic separation was achieved using a C18 column with a mobile phase consisting of water and acetonitrile containing 0.1% formic acid. Detection was performed using electrospray ionization. Quantification was based on external calibration curves with coefficients of determination greater than 0.99. As shown in Table S1, measured concentrations ranged from 87.0% to 95.8% of nominal concentrations, remaining within the ±20% acceptance range recommended by OECD 211 for semi-static exposure systems. Therefore, nominal concentrations were used for reporting and statistical analysis.

**Table S2** Primer sequence of this study

| Gene | Primer (5’-3’) | References |
| --- | --- | --- |
| *Cytochrome P450 360A8 (CYP360A8)* | Forward: TCGGCGAGATTTCACAGT  Reverse: GCACATTCGGTTATCAAGAC | Aksakal and Arslan 2020 |
| *Cytochrome P450 314*  *(CYP314)* | Forward: ACTATGTATGGACTTCCCTGGTG  Reverse: TTATCGCGGGTGTCAACG |  |
| *Cuticle Protein (CUT)* | Forward: AGCCAGTGGAACTACG  Reverse: TCCAGCATCATCAGCG |  |
| *Double Sex and Mab-3 Related Transcription Factor (DMRT93B)* | Forward: TAATCCCAATAACAGCAACGTG  Reverse: CTGCCGATGTTAGTCTTGAACA |  |
| *Glutathione S-Transferase (GST)* | Forward: GGGAGTCTTTTACCACCGTTTC  Reverse: TCGCCAGCAGCATACTTGTT |  |
| *Hormone Receptor 96 (HR96)* | Forward: GTCTGGGAAAGTTTGTGGAGTCT  Reverse: GAACCTGCGTGAACAGCATCTA |  |
| *P-glycoprotein (P-GP)* | Forward: CCACTTGCGTTCAACTTCTTC  Reverse: TTCGCCGATTGATGTTCC |  |
| *Vitellogenin (VTG)* | Forward: AGCGAATCCTACACCG  Reverse: CGACGAAGCTCAGCAA |  |
| *β-actin* | Forward: GCCCTCTTCCAGCCCTCATTCT  Reverse: TGGGGCAAGGGCGGTGATTT |  |

| Group | CT |
| --- | --- |
| Control | 22.22 |
| Control | 21.87 |
| Control | 22.15 |
| LUF 1 | 22.41 |
| LUF 1 | 21.02 |
| LUF 1 | 21.02 |
| LUF 2 | 21.13 |
| LUF 2 | 21.28 |
| LUF 2 | 20.8 |
| LUF 3 | 20.79 |
| LUF 3 | 21.06 |
| LUF 3 | 21.45 |

**Table S3** CT values of the β-actin gene

The Ct values of β-actin were compared across all experimental groups using one-way ANOVA. No statistically significant differences were observed (p > 0.05), indicating stable β-actin expression under the experimental conditions.


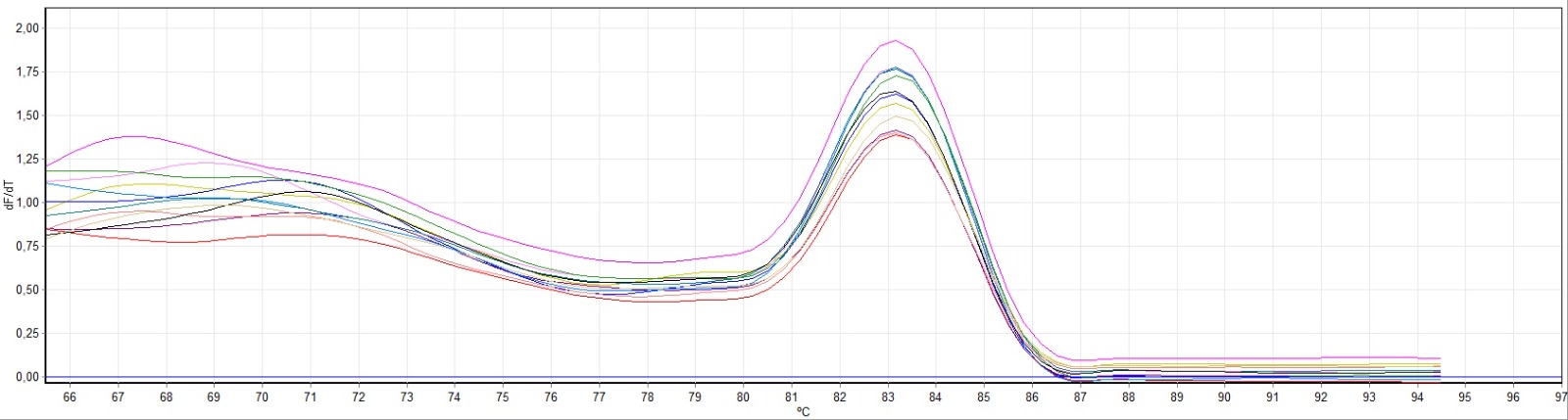


**Fig. S1** Representative melting curve of β-actin amplification in qRT-PCR analysis

Melt curve analysis showed a single, sharp peak for β-actin with consistent melting temperature across all samples, confirming specific amplification without primer-dimer formation.

**References**

Aksakal FI, Arslan H (2020) Detoxification and reproductive system-related gene expression following exposure to Cu(OH)_2_ nanopesticide in water flea (*Daphnia magna* Straus 1820). Environmental Science and Pollution Research 27, 6103–6111. <https://doi.org/10.1007/s11356-019-07414-x>
